# Impacts of Real-Time Aging on Kaolinite-Based Geopolymers in Ambient and Immersion Conditions

**DOI:** 10.3390/ma19112325

**Published:** 2026-06-01

**Authors:** Mazen Alshaaer, Juma’a Al-Kafawein, Sultan Almuaythir, Jan Wastiels

**Affiliations:** 1 Physics Department, College of Sciences and Humanities, Prince Sattam bin Abdulaziz University, Al-Kharj 11942, Saudi Arabia; 2 Chemistry Department, Collage of Science, Mu’tah University, Al-Karak 61710, Jordan; jkafawein@mutah.edu.jo; 3 Department of Civil Engineering, College of Engineering in Al-Kharj, Prince Sattam bin Abdulaziz University, Al-Kharj 11942, Saudi Arabia; s.alhomair@psau.edu.sa; 4 Department Mechanics of Materials and Constructions, Vrije Universiteit Brussels (VUB), Pleinlaan 2, 1050 Brussels, Belgium; jan.wastiels@vub.be

**Keywords:** uncalcined kaolinite, geopolymer, real-time aging, immersion, mechanical performance

## Abstract

This study explores the real-time aging of a 15-year-old uncalcined kaolinite-based geopolymer (UKG). The significance of this research lies in the fact that uncalcined kaolinite-based geopolymer is a relatively new material tailored for diverse applications, including construction, water treatment, and waste stabilization. While some studies have investigated its durability through accelerated tests, observing its aging over 15 years is essential for its commercial use and field deployment. The specimens were prepared from kaolinite, silica sand, sodium hydroxide, and water. The mixture was molded, compacted, and cured at 80 °C for 24 h to produce a stable geopolymer. Some samples were stored under ambient conditions, while others were immersed; both groups were left for 15 years. After this period, tests evaluated their mechanical, physical, and microstructural properties using XRD, EDS, and SEM. The samples stored under ambient conditions exhibited properties comparable to those of the unaged specimens. In contrast, the immersed samples were unstable, experienced mass loss, showed a sharp decline in strength, and displayed significant microstructural and phase changes. This study suggests adding an extra curing step, such as steaming (hydrothermal) or immersion in alkaline solutions, to enhance the long-term stability of geopolymer binder under immersion conditions.

## 1. Introduction

Geopolymers are a relatively new type of material with evolving properties and uses. Researchers have discovered that geopolymerization transforms aluminosilicates, such as clays, into aluminosilicate polymers called geopolymers [1,2]. These materials are composed of a three-dimensional, amorphous network primarily formed by the polymerization of aluminosilicate monomers in an alkaline environment. Much research has focused on exploring their mechanisms and chemistry [3,4]. It is believed that geopolymerization involves the polycondensation of hypothetical monomers, especially orthosilicate ions, leading to solid, stable products similar to natural minerals such as hydroxysodalite, feldspathoids, or zeolites [5]. The microstructure of geopolymers can be either amorphous, microcrystalline, or a combination of both, consisting of SiO_4_ and AlO_4_ tetrahedra linked through shared oxygen atoms. When aluminum bonds with oxygen, it acquires a negative charge that is neutralized by cations such as Na^+^, K^+^, Li^+^, Ca^2+^, Ba^2+^, NH_4_^+^, and H_3_O^+^ [6].

Most geopolymers form when suitable aluminosilicates are alkali-activated, yielding three-dimensional polymer networks [7]. Common sources of aluminosilicates include industrial by-products such as slag [8], fly ash [9], metakaolin [10], red mud [11], silica fume [12], and rice husk ash [13]. Alkali activators are usually compounds based on sodium or potassium, often combining KOH or NaOH with K_2_SiO_3_ or Na_2_SiO_3_ [2]. To enhance workability and reactivity, many aluminosilicates undergo thermal activation, such as processing into metakaolin, or incorporate industrial byproducts such as fly ash or blast furnace slag. Some studies examine the use of uncalcined kaolinite with NaOH for geopolymer production [14,15,16].

Life cycle assessments indicate that selecting different alkali–silicate activators significantly affects environmental impact; substituting them with KOH or NaOH can reduce this impact. Additionally, uncalcined naturally occurring aluminosilicates, such as kaolinite, can help lower CO_2_ emissions without thermal processing [17]. A geopolymer made from uncalcined kaolinite is an excellent building material, especially in developing countries, due to its many benefits. It uses an inexpensive, widely accessible precursor—uncalcined kaolinite. When combined with an alkali activator, it can be shaped and then heated at 80 °C [14,18,19,20]. Despite its benefits, the use of kaolinite as a precursor remains limited because it reacts less efficiently than materials like fly ash or metakaolin. However, processing kaolinite requires less energy than processing metakaolin. A small amount fully reacts with alkali to create binders, while larger amounts serve as partial fillers, which can weaken the structure [21].

One potential use for geopolymers is as a construction material. Uncalcined kaolinite-based geopolymers exhibit attractive mechanical properties, although several studies show that their strength drops by about 60% after water immersion [21]. For example, geopolymer produced from uncalcined Jordanian kaolinite exhibits a compressive strength of 41 MPa under dry conditions and 23 MPa after water immersion [22]. Functional untreated kaolinite-based geopolymers were introduced for both construction and water purification purposes [23]. Other studies have prepared geopolymers from untreated kaolinite and natural zeolite for the stabilization and solidification of heavy metals in kaolin/zeolite-based geopolymers [24].

Although kaolinite-based geopolymers, a relatively new material, have been used in many applications, there are few studies on their durability. One of these studies investigated the durability of alkali-activated products synthesized by activating untreated kaolinitic clay with a sodium hydroxide solution [22]. According to this study, the geopolymers underwent long-term durability tests under various environmental and chemical conditions. The specimens performed well in multiple environments, including ambient air, de-ionized water, wet–dry cycles, seawater, and sodium sulfate solutions. These samples maintain stable strength across all these conditions. Therefore, the significance of this study lies in evaluating uncalcined kaolinite-based geopolymers, a relatively new material, under real-time aging conditions in both ambient and immersion environments. The samples were aged for 15 years in ambient and immersed conditions. The microstructural and physical changes in the samples during this extended period were measured, analyzed, and evaluated.

## 2. Materials and Methods

### 2.1. Materials and Chemicals

Geopolymers were produced using uncalcined kaolinite, silica sand, and a sodium hydroxide-based alkaline activator. Kaolinite (Merck, Darmstadt, Germany), with purity > 99% and grain size < 60 µm, serves as the solid reactant and supplies Al ions.

Silica sand, from International Silica Industries Co., Amman, Jordan, is a common inert filler for cementitious materials. It was washed with diluted HCl (0.1 N) for 10 min to remove impurities and sieved to ~400 μm. The NaOH solution, used as an alkaline activator to dissolve aluminosilicate phases, was prepared by dissolving 98% pure NaOH flakes (Scharlau, Sentmenat, Spain) in distilled water.

### 2.2. Synthesis of Geopolymers’ Specimens

The amounts of kaolinite and sand were weighed and blended using a mechanical overhead mixer. NaOH solution was gradually added to the kaolinite/sand mixture and stirred for 2 min at 100 rpm, then for 10 min at 200 rpm to ensure thorough homogenization. The mixture was then placed into a stainless-steel cylinder mold (60 mm height, 25 mm diameter) and compacted at 15 MPa using a hydraulic press. After removing the mold, the sample was weighed. At least three samples were prepared from each mixture. The samples were oven-dried at 80 °C for 24 h in a ventilated oven, then cooled to room temperature. The mixture used to prepare the samples has mass fractions of 100:100:28:22 for kaolinite, silica sand, water, and NaOH, respectively (Table 1). These ratios are based on workability, the clay’s plasticity limit, and the estimated degree of geopolymerization, as previously explained [21,25].

**Figure 1:**
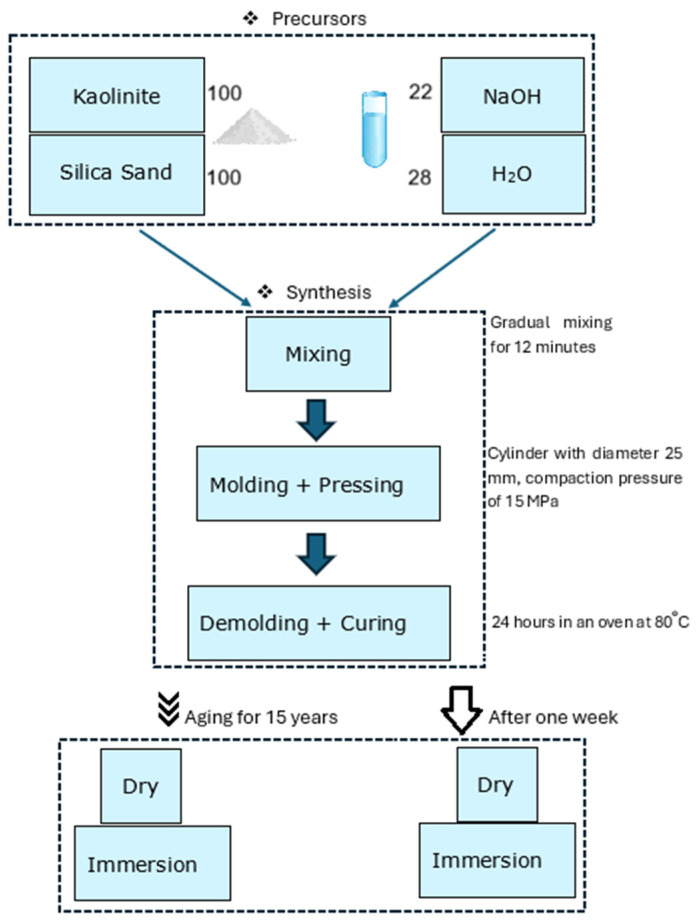
Experimental procedure for preparing kaolinite-based geopolymers.

To assess the stability and durability of uncalcined kaolinite-based geopolymers (GK), three samples were kept at ambient conditions for 7 days, while the other three were submerged in distilled water for the same period. Additionally, three samples were stored at ambient conditions for 15 years, while three others were continuously immersed in distilled water for that period. The samples were organized into four series, as shown in Table 1, and the experimental procedure is depicted in Figure 1.

Ambient conditions refer to the indoor environment in Amman, where the samples were stored for 15 years, with annual temperature variations of approximately 3 °C to 33 °C and relative humidity of 36% to 69%. No artificial conditioning, whether cooling or heating, was used in the storage room during this period.

### 2.3. Characterization and Evaluation of Geopolymer Properties

The phase composition, along with the physical, mechanical, and microstructural properties of the geopolymers, was analyzed under both dry and immersion conditions. Tests were conducted on unaged specimens over 7 days and on specimens aged for 15 years. Different equipment was used to test the unaged samples at the University of Jordan, Jordan, and the aged samples at Prince Sattam bin Abdulaziz University, Saudi Arabia. Table 2.

The compression test was performed using a universal testing machine, with load applied and increased at a displacement rate of 2 mm/min. Three dried specimens and three immersed specimens were subjected to a compressive strength test. X-ray diffraction (XRD) analysis was performed on powdered samples to identify the major crystalline phases and any potentially newly formed phases. The XRD patterns were measured from 5° to 80° 2θ at a scan rate of 2°/min. MATCH! Software (version 4) was used to perform Rietveld refinements of the materials produced (Version 4, Crystal Impact, Bonn, Germany). The morphology of the specimens was studied using a Scanning Electron Microscope. The samples were precoated with platinum under an argon atmosphere. The attached Energy-dispersive X-ray spectroscopy (EDX) was used for elemental analysis of the samples. Further information about the models and the manufacturers of the instruments is reported in Table 2.

Water absorption for each series was measured according to the following equation:

$$Absorption\%=\left(\frac{M_{w}-M_{d}}{M_{d}}\right)\times 100\%$$

where M_d_ represents the mass of dried specimens after 80 °C drying for 7 days, and M_w_ indicates the mass of specimens saturated with water, after immersion in distilled water for 7 days.

## 3. Results and Discussion

### 3.1. Phase Composition and Microstructural Analysis

#### 3.1.1. GK-d Geopolymer

**Figure 2:**
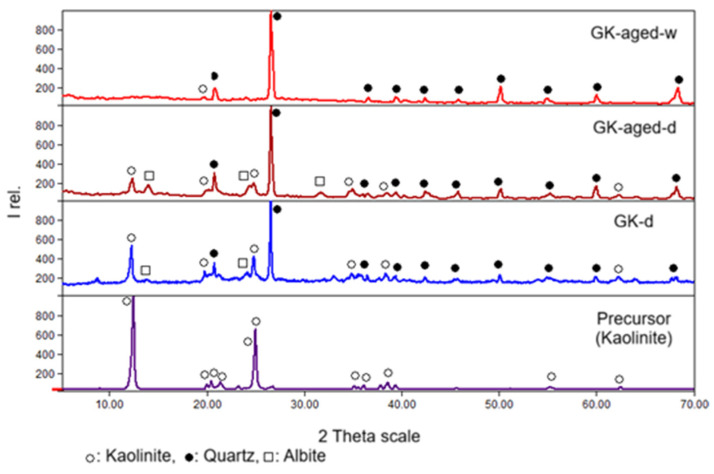
XRD patterns of precursors, unaged, and aged geopolymers.

The XRD patterns of kaolinite (precursor), and both unaged and aged geopolymers under ambient and immersed conditions are shown in Figure 2. The XRD patterns of the geopolymer (GK-d) indicate that during alkali geopolymerization, the kaolinite peaks decrease as albite forms. This mineral transformation is accompanied by the development of an amorphous structure of sodium aluminosilicates, as noted in the XRD hump between 20° and 40° [20,21]. Quartz peaks, which serve as fillers, are visible in the XRD patterns corresponding to GK-d.

**Figure 3:**
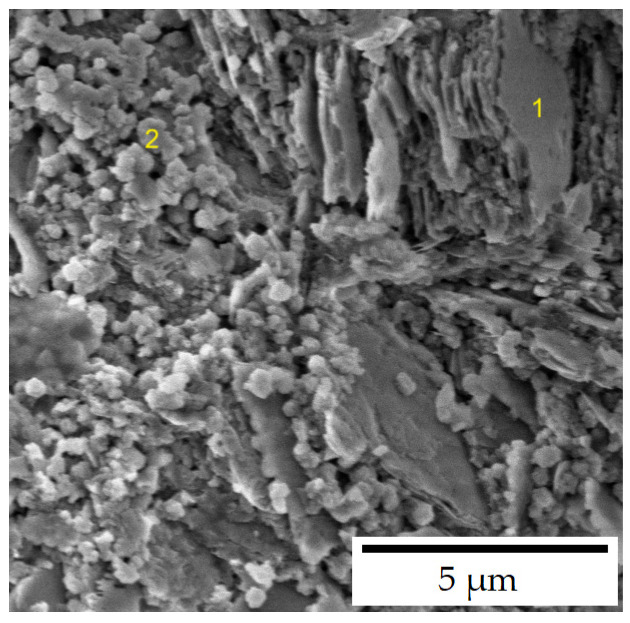
SEM image of unaged geopolymer (GK-d); point (1), kaolinite layers, and point (2), geopolymer products.

The microstructure of GK-d geopolymers, as shown in the SEM image (Figure 3), exhibits the coexistence of geopolymer binder and partially unreacted kaolinite layers. These results agree with the X-ray analysis in Figure 2, which shows the kaolinite layers, Figure 3—point (1). Additionally, the products of the geopolymerization reactions appear as nanosized granules (Figure 3—point (2)) embedded between the unreacted kaolinite layers, as a geopolymer binder.

**Figure 4:**
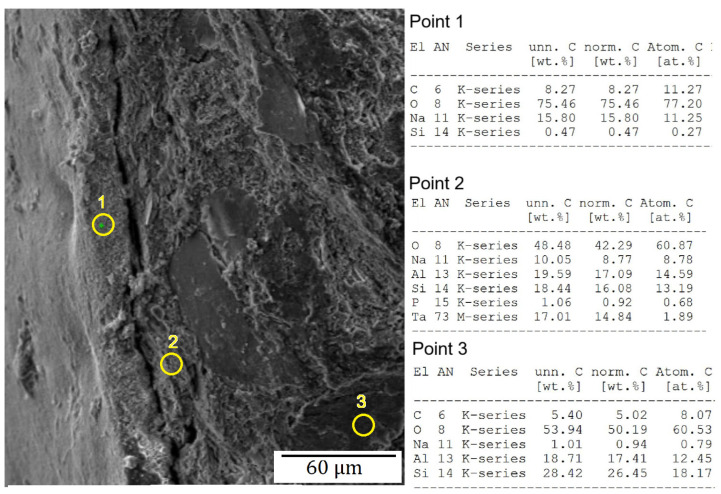
SEM image and EDS analysis of the surface and subsurface of unaged geopolymer.

To assess the microstructural uniformity of GK-d, surface and subsurface analyses were conducted using SEM and EDS. The SEM image (Figure 4, point 1) reveals a thin (~20 µm) coating on the geopolymer surface. EDS results show that this layer contains Na and C, indicating it is likely Na_2_CO_3_. During curing, water evaporates, leaving unreacted salts such as NaOH on the surface. These salts react with atmospheric CO_2_ (carbonation) to form Na_2_CO_3_. This Na-based salt is water-soluble and basic, with a pH around 11. Under dry conditions, Na_2_CO_3_ has little effect on geopolymer performance as a construction material, but in the presence of water, it can cause problems. Dissolved Na_2_CO_3_ can raise the pH of pore water, promoting alkaline degradation of the geopolymer components, especially kaolinite. EDS analysis shows the average Si/Al/Na molar ratio in the subsurface geopolymer binder (point 2) is 1/0.9/1.5. This sodium aluminosilicate matrix is similar to that of the geopolymer binder. The ratios in partially transformed kaolinite layers are higher than in pure kaolinite, with Si/Al at 1.46 versus 1. This indicates that unreacted kaolinite is altered by geopolymerization, partially releasing Al ions that contribute to the geopolymer binder.

#### 3.1.2. GK-Aged-d Geopolymer

**Figure 5:**
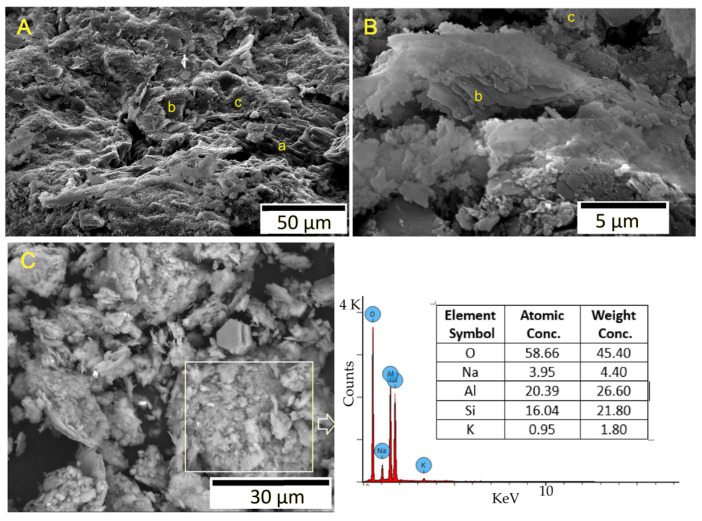
SEM and EDS analysis of GK-aged-d specimens: surface morphology (**A**), high-magnification surface detail (**B**), and EDS analysis (**C**); point (a), quartz, point (b), kaolinite layers, and point (c) geopolymer binder.

As a result of aging for 15 years under ambient conditions, the XRD patterns show a reduction in the peaks corresponding to kaolinite. New peaks corresponding to albite are observed at 31.84° (Figure 2). The SEM images in Figure 5 illustrate microstructural morphology and the EDS analysis of the GK-aged-d specimens. Fine grains of quartz, Figure 5A (point a), are observed in the microstructure of GK-aged, along with kaolinite layers, Figure 5A,B (point b), and the geopolymer binder, Figure 5A,B (point c). These three microstructural phases are typical features of kaolinite-based geopolymer [20]. Compared with unaged specimens (Figure 4), EDS analysis (Figure 5C) shows that the elemental composition of the geopolymer matrix was altered by long-term aging, with the Si content increasing relative to Al and N.

#### 3.1.3. GK-Aged-w Geopolymer

**Figure 6:**
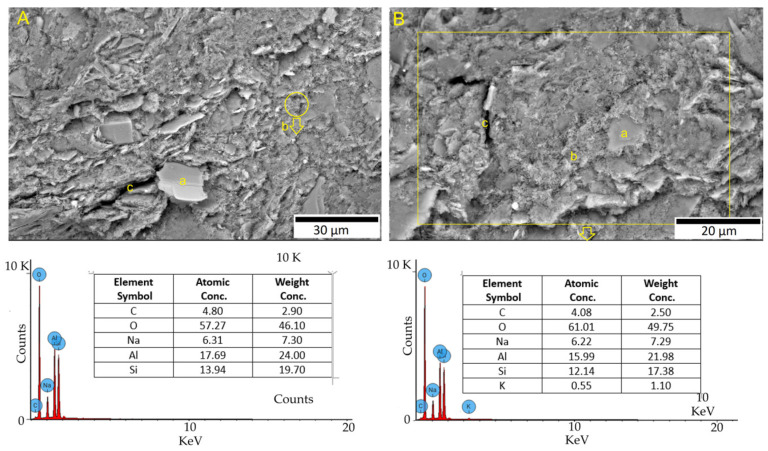
SEM and EDS analysis of GK-aged-w specimen, point EDS Analysis (**A**), and elemental mapping (EDS) (**B**); point (a), kaolinite layers, point (b), geopolymer binder, and point (c) voids.

Long-term aging of the kaolinite-based geopolymer for 15 years (GK-aged-w) under immersion conditions leads to a decrease in most peaks associated with kaolinite and albite, as shown in Figure 2. SEM analysis of GK-aged-w, shown in Figure 5, confirms the presence of kaolinite layers (Figure 6, point (a)) that are randomly dispersed within the geopolymer matrix. Additionally, a very fine matrix or amorphous phase (Figure 6, point (b)) is observed along with the formation of micro-voids (Figure 6, point (c)). EDS analysis reveals that this matrix is composed of aluminum sodium silicates. The overall element mapping in GK-agedw, Figure 6B at point (a), indicates the presence of the three main elements—Al, Si, and Na—though the sodium content is higher compared to GK-aged-d.

#### 3.1.4. Analysis of Phase and Microstructural Changes as a Result of Long-Term Aging

In summary, long-term aging under ambient conditions has only a limited impact on the microstructure and phase composition of kaolinite-based geopolymers, as shown by Rietveld refinement of powder X-ray diffraction (Table 3). There is a slight decrease in the amorphous phase content from 33.9% to 31.9% due to aging. In general, geopolymer binders have an amorphous structure, so this phase significantly influences the effects of aging on structural integrity. The three crystalline phases—quartz (filler), kaolinite (precursor), and albite (product)—are also present in the GK-aged-d, although the albite content decreases from 23% to 4%. The main processes during long-term indoor aging include seasonal variations in temperature, from about 3 °C to 33 °C, and relative humidity, from 36% to 69%. These changes can lead to cyclic absorption and release of pore moisture. Pore moisture dissolves residual alkaline salts, maintaining a high pH that continues to react with kaolinite. Consequently, cyclic pore moisture causes changes in the geopolymer binder, notably a substantial long-term reduction in albite.

Prolonged aging in distilled water results in the leaching of residual alkaline salts, raising the pH from 7 to 13 and causing ongoing alkaline corrosion of the kaolinite. Over time, this mineral structure becomes deformed and transitions into an amorphous form. Consequently, the geopolymer structure weakens and begins to leach, evidenced by the random voids observed in the microstructure.

**Figure 7:**
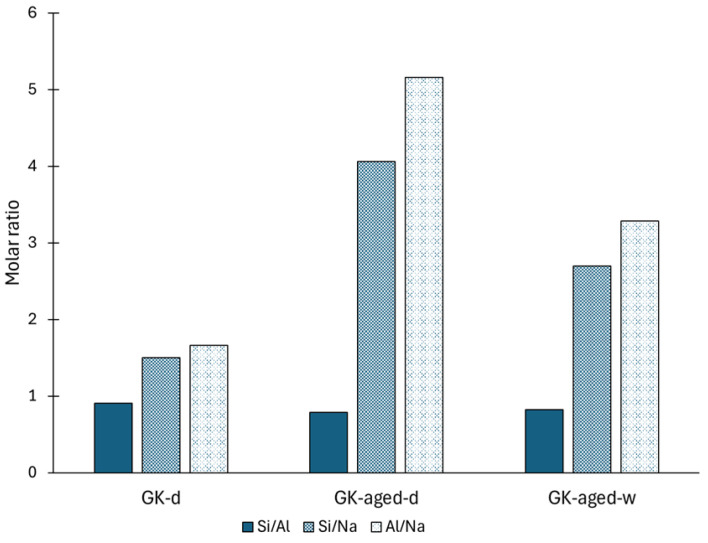
Molar ratios of the geopolymer matrix (binder): unaged, aged under ambient conditions, and aged under immersion.

The composition of the amorphous phase in the geopolymer binder for the three series—GK-d, GK-aged-d, and GK-aged-w—is shown in Figure 7. This analysis uses EDS data from Figure 4, Figure 5 and Figure 6. Notably, there is a significant decrease in Na levels in the aged samples exposed to ambient (GK-aged-d) and immersion (GK-aged-w) conditions, compared to Al and Si. This suggests a steady supply of Si and Al to the geopolymer matrix driven by ongoing reactions under ambient conditions. While similar reactions occur in water (GK-aged-w), leading to reduced Si and Al concentrations, likely due to partial degradation and leaching of the contents.

### 3.2. The Impact of Long-Term Aging on the Physical and Mechanical Characteristics of the Geopolymers

**Figure 8:**
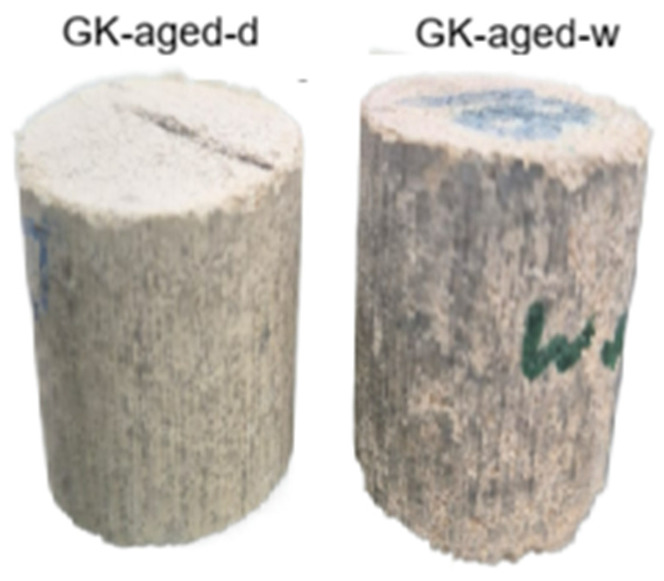
Photos of GK-aged-d and GK-aged-w.

**Figure 9:**
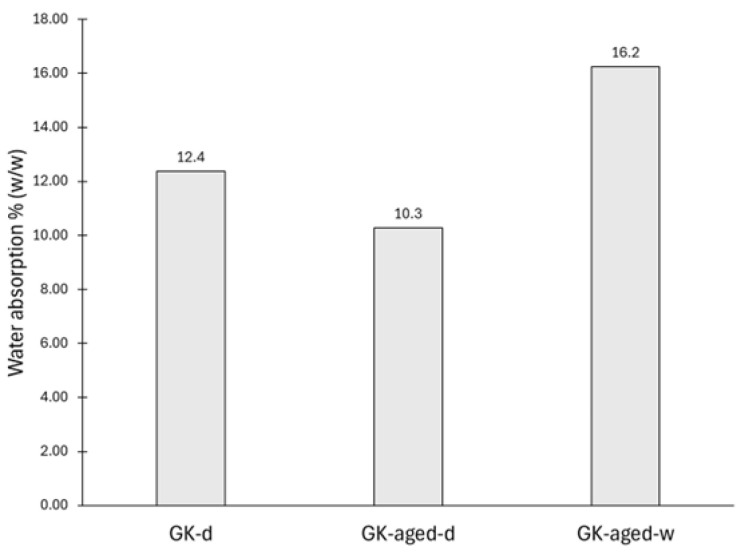
Water absorption of GK-d, GK-aged-d, and GK-aged-w.

Both GK-aged-d and GK-aged-w demonstrate a good appearance, Figure 8, with very low linear shrinkage (<0.1%). The bulk density of GK-aged-d (1.96 g/cm^3^) is similar to that of GK-d specimens (1.95 g/cm^3^), Figure 9. In contrast, GK-aged-w specimens exhibit a significant decrease in bulk density, measuring 1.68 g/cm^3^. These findings indicate that long-term immersion aging results in substantial mass loss.

**Figure 10:**
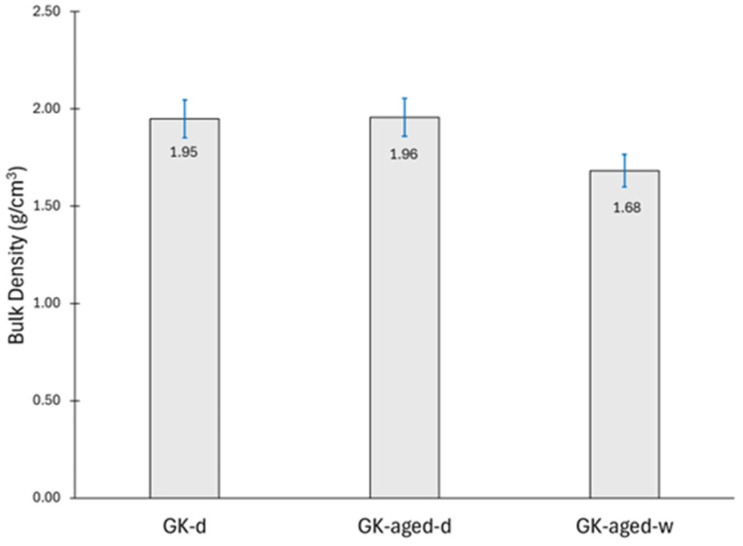
Bulk density of GK-d, GK-aged-d, and GK-aged-w.

The water absorption of geopolymers drops from 12.4 to 10.3 when aged under ambient conditions (GK-aged-d), as shown in Figure 10, mainly due to reduced open porosity caused by carbonation and ongoing reactions, as previously discussed. Conversely, the water absorption of GK-aged-w specimens rises sharply from 12.4% to 16.2% (*w*/*w*). This notable increase aligns with results from bulk density and microstructural analyses. After long-term immersion, GK-aged-w gradually loses mass, resulting in high open porosity and voids.

**Figure 11:**
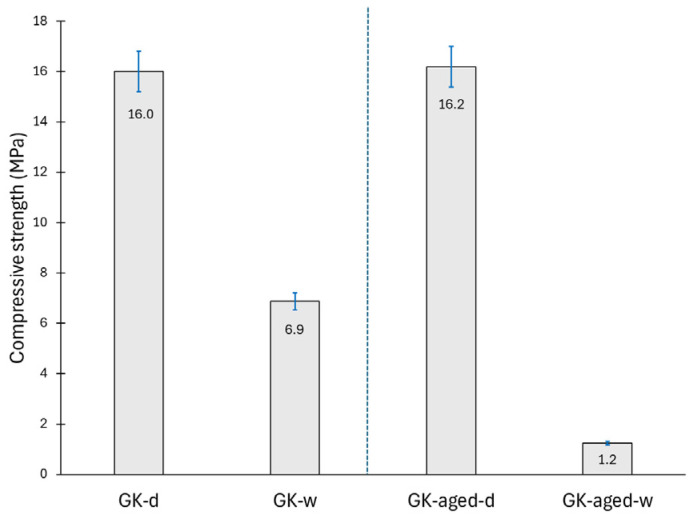
Compressive strength of unaged specimens and aged specimens under ambient and immersion conditions, Table 1.

The compressive strength of GK-d, GK-w, GK-aged-d, and GK-aged-w is presented in Figure 11. The compressive strength of the geopolymers, GK-w, decreases by 57%, from 16 MPa to 6.9 MPa, after 1 week of immersion in water, a finding consistent with previous research [14,20]. Nevertheless, after fifteen years of aging under ambient conditions, a slight increase in compressive strength to 16.2 MPa is observed. Conversely, the aged specimens subjected to immersion, GK-aged-w, exhibit a significant reduction in compressive strength to 1.2 MPa. These notable failures in material strength after prolonged underwater aging are consistent with the previous discussion on bulk density, water absorption, and microstructural analysis, in which the mass loss of the specimens destabilizes the structure and mechanically weakens it.

This study shows that the uncalcined kaolinite-based geopolymer maintained its mechanical performance for 15 years under dry conditions. Minor microstructural changes were observed, similar to those observed in other cement-like materials. These changes were caused by interactions between alkali salt residues and the environment, as well as by seasonal fluctuations in pore moisture. Despite these microstructural changes, the samples’ physical properties, such as density and water absorption, remained stable over time.

This material’s performance is notably affected by its strength reduction after prolonged immersion, leading to changes in density and porosity. Initial analyses indicated a mass loss of approximately 14%, with the displaced material entering the water and leaving behind a fragile structure characterized by open pores and sizable voids. The binding component underwent ongoing reactions, particularly between the alkalized water—due to leached alkaline salts—and kaolinite, as well as the geopolymer binder itself. These continuous transformations gradually degraded the material’s bonding, ultimately reducing its strength.

**Figure 12:**
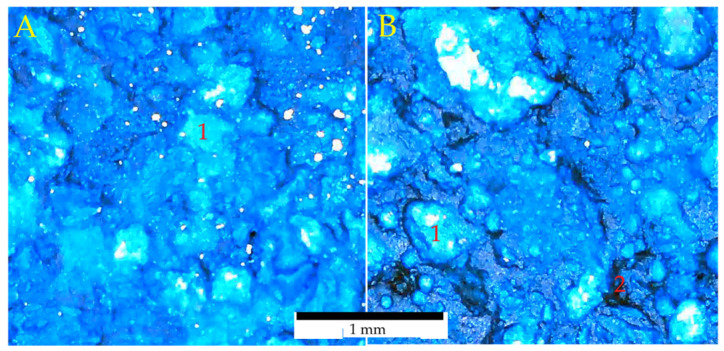
Optical image of the internal surface texture of the samples’ cross-section after ink coloring: GK-aged-d (**A**) and GK-aged-w (**B**), sand grains (point 1), and microchannels (point 2).

Figure 12 shows the internal surface textures of the GK-aged-d and GK-aged-w samples after ink coloring. The images reveal that extended immersion of GK-aged-w samples can form microchannel-like patterns (Figure 12B) that are not observed in GK-aged-d. These micrometer-wide channels develop over 15 years of aging under immersion conditions, starting at sand-grain boundaries and extending into the geopolymer matrix and unreacted kaolinite. This microchannel structure results from slow, long-term mass removal and erosion. Variations in channel irregularity are influenced by two factors: increasing solution alkalinity due to leaching and the heterogeneous composition of the samples, which leads to different reactions at microscale sites. These findings support the idea of mass loss over time and the related decline in mechanical performance after prolonged immersion aging.

Although it is not recommended to use these materials for long-term applications in water-rich environments, some solutions may strengthen and stabilize the geopolymer binder and transform a considerable amount of kaolinite during the main reaction, as it is the component most affected by the aquatic environment. So, the solution to this challenge may be to prepare the untreated kaolinite geopolymer using the two-phase geopolymerization method [21] to ensure that the most significant amount of kaolinite reacts during the main geopolymerization reactions, or to use the hydrothermal curing method [26].

### 3.3. Summary: Real-Time Aging of Kaolinite-Based Geopolymers Under Ambient and Immersion Conditions

Long-term ambient aging has little effect on the microstructure and phase makeup of kaolinite-based geopolymers. The amorphous content slightly declines from 33.9% to 31.9%. Since geopolymer binders are mainly amorphous, this impacts how aging influences their structural stability. Crystalline phases like quartz, kaolinite, and albite are present; notably, albite decreases from 23% to 4%. Indoor aging involves seasonal temperature changes (3 °C to 33 °C) and humidity shifts (36% to 69%), causing cyclic moisture absorption and release that dissolves residual salts and sustains a high pH (~13). This ongoing process reacts with kaolinite, gradually reducing albite. The external surface also experiences carbonation. After 15 years, there was a slight increase in compressive strength. The uncalcined kaolinite-based geopolymer preserved its mechanical properties under ambient conditions. Minor microstructural changes—due to alkali salt interactions and seasonal moisture fluctuations—were observed, but properties like density and water absorption remained stable.

**Figure 13:**
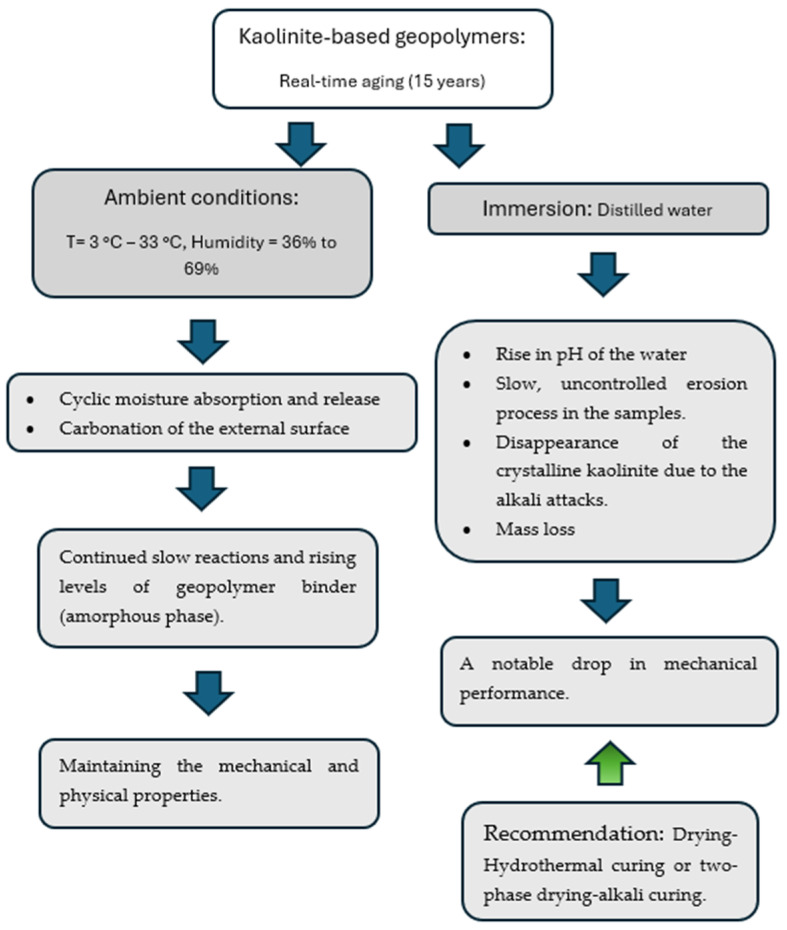
Real-time aging process of kaolinite-based geopolymer.

Extended aging in water causes residual salts to leach out, raising pH and leading to alkaline corrosion of kaolinite. Over time, this deforms the mineral structure, weakening the kaolinite-based geopolymer and causing microstructural voids. Water reduces Si and Al, likely due to partial degradation, though specimens remain visually intact. They exhibit lower bulk density, indicating significant mass loss and reduced strength, reflecting microstructural deterioration. About 14% of mass was lost, with displaced material entering the water, creating a fragile, porous structure (Figure 13). These materials are unsuitable for long-term exposure to water, but treatments such as two-phase geopolymerization or drying-hydrothermal curing can improve strength and stability by promoting kaolinite transformation.

## 4. Conclusions

This study investigated the aging of uncalcined kaolinite-based geopolymers over 15 years under both ambient and immersion conditions. Results showed that, under ambient conditions, these geopolymers maintained their mechanical properties throughout the period. Slight microstructural changes, similar to those observed in other cementitious materials, occurred due to reactions involving alkali-salt residues, environmental factors, and seasonal variations in pore moisture. Despite these microstructural modifications, key physical properties such as density and water absorption remained unaffected.

In contrast, extended immersion markedly reduced performance, resulting in lower strength and density and increased water absorption and voids. Initial findings indicated approximately 14% mass loss, with leached materials forming a fragile, porous structure with large voids and internal channels. The alkalized water, due to the leaching of residual alkali from the samples, continued to react with kaolinite and the geopolymer binder, gradually weakening the structural bonds and diminishing strength.

Although long-term use in water-rich environments is not recommended, approaches like enhancing the geopolymer binder’s stability or increasing kaolinite’s reactivity could help delay deterioration. Curing methods such as two-phase geopolymerization, drying-alkali, or hydrothermal, drying–hydrothermal, may promote more complete reaction of kaolinite during initial geopolymerization, thereby improving durability.

## Figures and Tables

**Table 1:** Composition and pre-testing treatments of specimens used in this study.

	Composition (Mass Fraction)					
ID	Kaolinite	Silica Sand	Water	NaOH	Treatment	Aging Period
GK-d	100	100	28	22	Ambient conditions ^1^	7 days (unaged)
GK-w	100	100	28	22	immersion	7 days (unaged)
GK-Aged-d	100	100	28	22	Ambient conditions	15 years
GK-Aged-w	100	100	28	22	immersion	15 years

^1^ According to the indoor conditions in Amman.

**Table 2:** Testing and characterization instruments are applied to both unaged and aged specimens.

Characteristics	Instrument	Unaged Specimens	Aged Specimens for 15 Years
Compressive strength	Universal testing machine	CONTROLS testing machine (Model T106), Liscate, Italy	(HD-B615-S, Haida International Equipment Co., Ltd., Dongguan, China
Phase composition	XRD	diffractometer-6000 (Shimadzu), Kyoto, Japan	Rigaku Ultima IV XRD diffractometer-6000, Akishima, Japan
Microstructure	SEM-EDS	Quanta Inspect F50, FEI Company, (coating with platinum), Eindhoven, The Netherlands	Quanta Inspect F50, FEI Company, (coating with gold), Eindhoven, The Netherlands

**Table 3:** Rietveld refinement of powder X-ray diffraction (XRD) data, Figure 2, based on the MATCH! Software analysis, version 4.

	DoC	Crys. Phases	Crystal System	%	Space Group	Unit Cell Constants (Å)	R. Chi^2^	Unit Cell V. (Å^3^)	**Crystallite Size (Å)**
Kaolinite	63.0	Kaolinite	triclinic	100	C 1	a = 5.15, b = 8.94, c = 7.39, α = 91.93°, β = 105.05°, γ = 89.80°	0.7	304.659	402
Silica sand	85	Quartz	trigonal	100	P 32 2 1 S	a = 4.92 c = 5.41	2.8	113.128	998
GK-d	33.9	Kaolinite	triclinic	33	C1	a = 5.15, b = 8.94, c = 7.39, α = 91.93°, β = 105.05°, γ = 89.80°	0.1	328.706	453
		Quartz	trigonal	44	P 31 2 1	a = 4.91, c = 5.40,		112.961	1187
		Albite	triclinic	23	C −1	a = 8.15, b = 12.87, c = 7.11, α = 93.52°, β = 116.46°, γ = 90.26°		665.906	270
GK-aged-d	31.9	Kaolinite	triclinic	42	P1	a = 5.17, b = 8.99, c = 7.35, α = 91.684°, β = 105.13°, γ = 89.76°	0.2	329.782	140
		Quartz	trigonal	54	P 32 2 1 S	a = 4.92, c = 5.41,		113.128	300
		Albite	triclinic	4	C −1	a = 8.15, b = 12.87y, c = 7.11, α = 93.52°, β = 116.46°, γ = 90.26°		665.906	154
GK-aged-w	23.1	Kaolinite	triclinic	1	P 1	a = 5.17, b = 8.98, c = 7.35, α = 91.68°, β = 105.13°, γ = 89.76°	0.1	329.782	x
		Quartz	trigonal	86	P 32 2 1 S	a = 4.91, c = 5.40,		112.739	275
		Albite	triclinic	13	C −1	a = 8.15, b = 12.87, c = 7.11, α = 93.52°, β = 116.46°, γ = 90.26°		665.906	x

## Data Availability

The original contributions presented in this study are included in the article. Further inquiries can be directed to the corresponding author.
